# Corneal ectasia, cerulean (blue dot) cataract with acute hydrops in a child with Down’s syndrome and hypothyroidism – a rare presentation

**DOI:** 10.3205/oc000215

**Published:** 2023-03-01

**Authors:** Bharat Gurnani, Kirandeep Kaur, Shivanand Narayana

**Affiliations:** 1 Cornea and Refractive Services, Aravind Eye Hospital and Post Graduate Institute of Ophthalmology, Pondicherry, India; 2 Cataract, Paediatric and Squint Services, Aravind Eye Hospital and Post Graduate Institute of Ophthalmology, Pondicherry, India

**Keywords:** Down’s syndrome, acute corneal hydrops, keratoconus, congenital cerulean cataract

## Abstract

Down’s syndrome or trisomy 21 is a genetic disorder caused by presence of all or a part of a third copy of chromosome 21. Keratoconus occurs in up to 15% of the adult population with Down’s syndrome. There is a close consortium between trisomy 21 and keratoconus (a conical ectatic protrusion of the cornea), and children with Down’s syndrome are also susceptible to developing thyroid disease, including hypothyroidism and thyrotoxicosis with exophthalmos. The authors describe a case report on acute corneal hydrops with congenital cerulean cataract in a patient with Down’s syndrome with hypothyroidism having bilateral advanced keratoconus. As per the detailed literature review, this is the first case of Down’s syndrome with hypothyroidism presenting with acute corneal hydrops.

## Introduction

Down’s syndrome (DS) is a congenital anomaly with enormous medical and social costs, caused by trisomy of the whole or a part of chromosome 21 [1]. It is a widespread genetic disease worldwide and the usual genetic cause of intellectual disabilities appearing in about 1 in 400–1500 newborns [2]. There is a close consortium between trisomy 21 and keratoconus (a conical ectatic protrusion of the cornea) [3], and children with Down’s syndrome are also susceptible to developing thyroid disease, including hypothyroidism [4] and thyrotoxicosis with exophthalmos [5]. Hypothyroidism is one of the most common endocrine problems in children with Down’s syndrome. Nearly 10% of children with Down’s syndrome have congenital or acquired thyroid disease. Hypothyroidism can lead to symptoms of fatigue, mental sluggishness, weight fluctuations and irritability [6]. 

Moreover, Keratoconus occurs in up to 15% of the adult population with Down’s syndrome [7]. Children with Down’s syndrome can develop cataracts, both congenital and acquired, particularly cerulean blue dot cataracts composed of amyloid-β deposits [8]. Here we report a case of a 19-year-old with Down’s syndrome and hypothyroidism that presented to us with bilateral advanced keratoconus, congenital cerulean blue dot cataract with acute corneal hydrops. As per our knowledge and detailed literature search revealed that this is the first case of Down’s syndrome with hypothyroidism that presented with acute corneal hydrops.

## Case description

**Figure 1:**
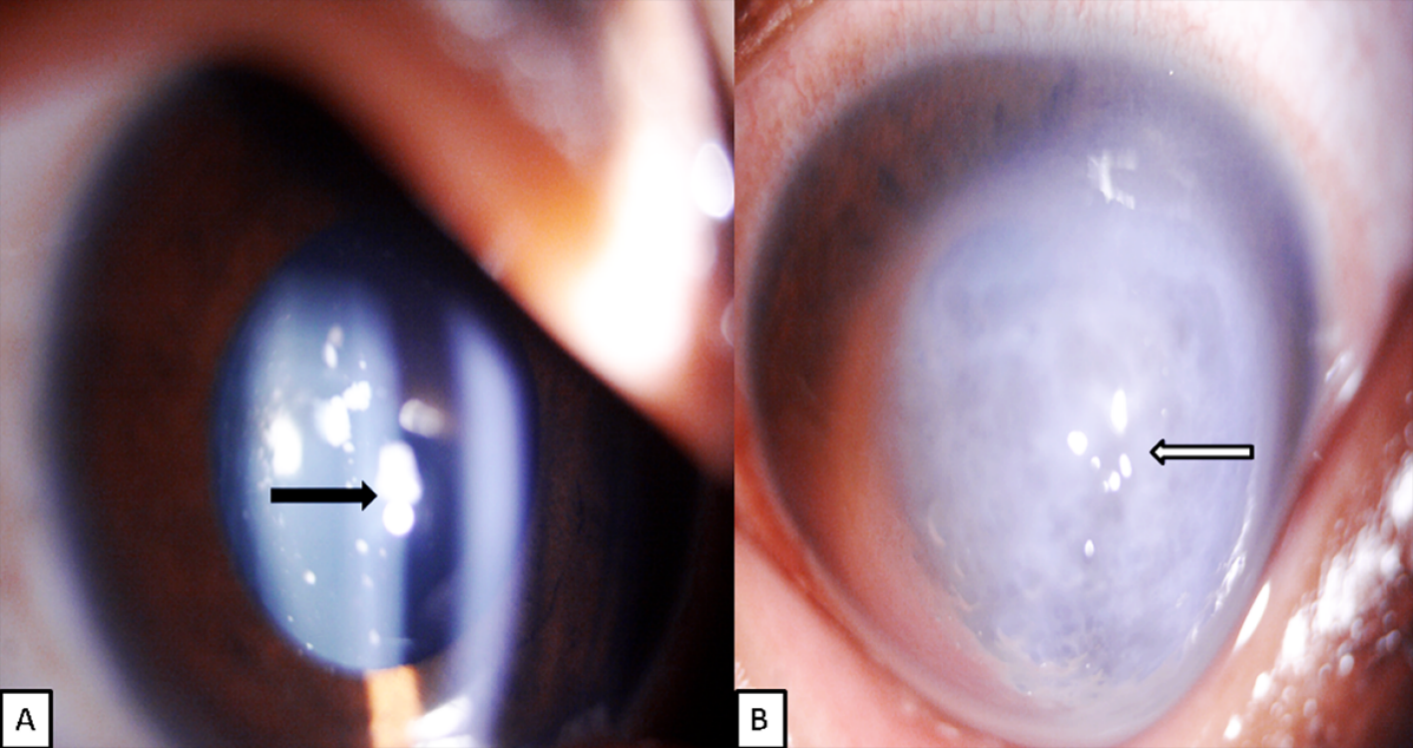
A: Image of the patient’s right eye depicting congenital cerulean (blue) dot cataract. B: Image of the patient’s left eye depicting acute corneal hydrops.

**Figure 2:**
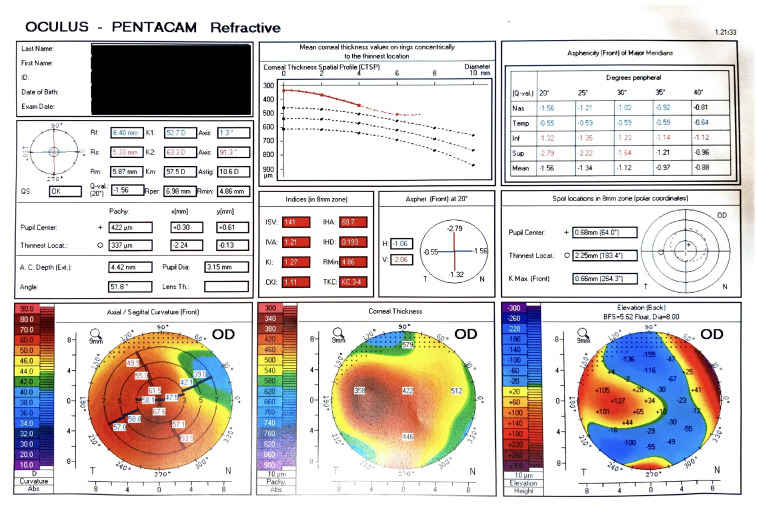
Image of the pentacam of the patient’s right eye depicting elevated K1 and K2 reading, thinnest pachymentry of 337 μm, paracentral and mild inferior steepening, anterior and posterior float elevation, altered indices and corneal thickness spatial profile suggestive of advanced keratoconus changes

A 19-year-old male with trisomy 21 was referred to our tertiary eye care centre with 10-day history of pain, watering, photophobia, and defective vision in left eye (LE), informant being the father. The best corrected visual acuity in right eye (RE) was 5/60 and in LE was hand movements. Anterior segment examination in right eye revealed Fleischer’s ring, conical protrusion of cornea with blue dot cataract (Figure 1A (Fig. 1)), and LE examination revealed circumcorneal congestion, conical protrusion of cornea with epithelial bullae with diffuse stromal edema and a break in Descemet membrane suggestive of acute corneal hydrops (Figure 1B (Fig. 1)). Pentacam evaluation of the right eye revealed advanced keratoconus with K1 –52.7 D, K2 –63.3 D and thinnest pachymetry value of 337 μm (Figure 2 (Fig. 2)). LE Pentacam was deferred in view of pain and photophobia. The patient was treated with topical antibiotics, cycloplegic and 5% sodium chloride eye drops in the LE. 

Detailed history revealed child was diagnosed with Down’s syndrome at birth. Hypothyroidism was confirmed through biochemistry at a tertiary care centre with {thyroid-stimulating hormone (TSH)>0.05 mU/litre, FT4 0.6 ng/dl, FT3 70 ng/dl}. 

**Figure 3:**
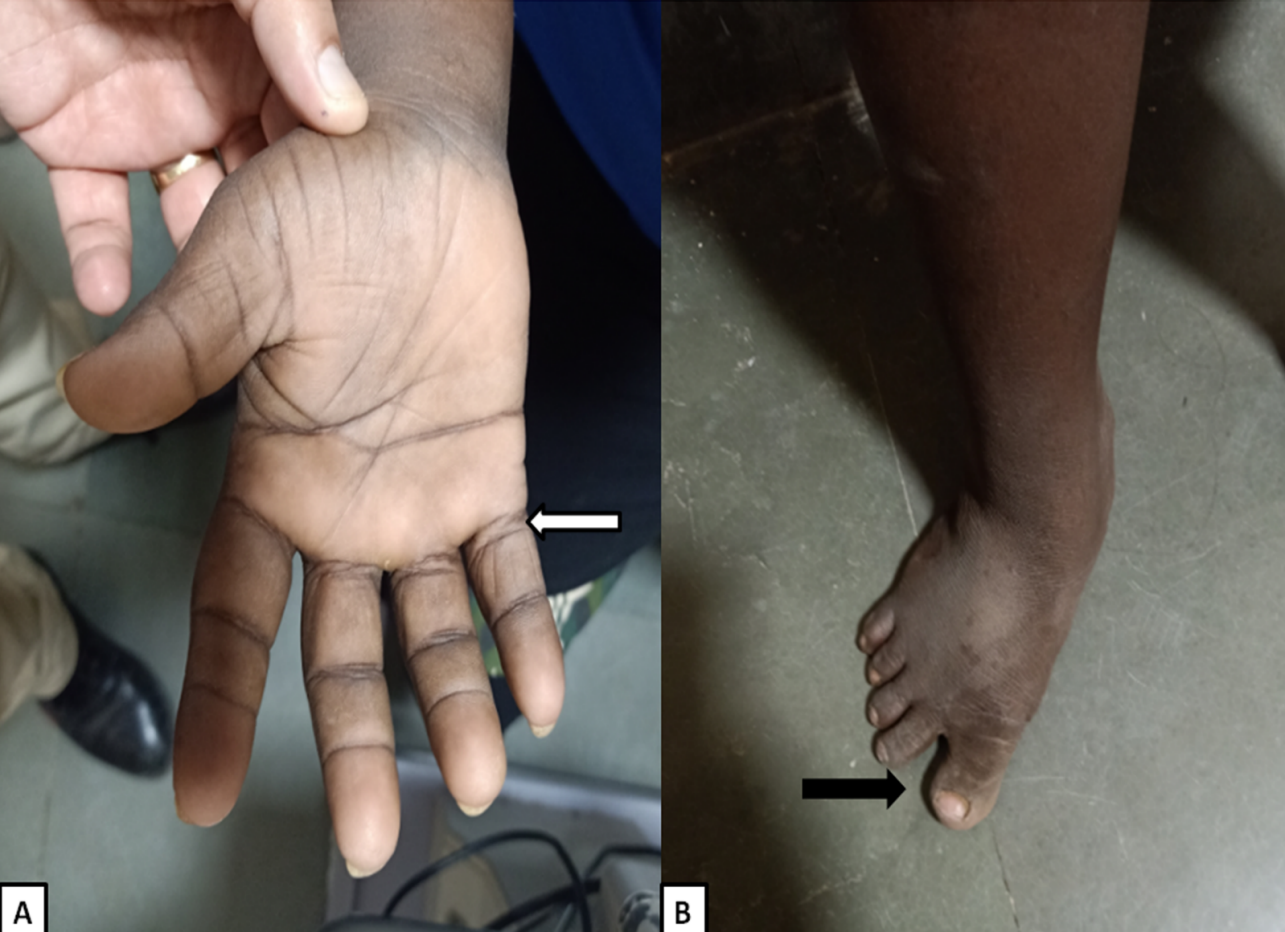
A: Image of the patient’s hand depicting short broad hands, short fifth finger, and single palmar crease. B: Image of the patient’s leg depicting widened gap between first and second toe (sandal gap).

General physical examination depicted flat facial profile, upward slant of eye, epicanthal folds, enlarged protruded tongue, excessive skin on nape of neck with short neck, abnormal shaped ears, short broad hands, short fifth finger, single palmar crease, widened gap between first and second toe (sandal gap) (Figure 3A/B (Fig. 3)), small bones, hyper flexibility and muscular hypotonia. The patient also had delayed motor milestones, learning difficulty, hearing loss, and small stature.

## Discussion

Advanced keratoconus has been a known association in children with Down’s syndrome. Acute hydrops has also been noted in children with keratoconus with trisomy 21 [9]. Imbalance of thyroid hormone can create abnormalities in structure, physiology and function of the eye [10]. Gatzioufas et al. highlighted in their article that thyroid gland dysfunction may be associated with keratoconus pathophysiology: for example, changes in maternal thyroid hormone levels during pregnancy can aggravate the progression of keratoconus [11]. Hence, it supports the assumption that the hypothyroidism was an additional contributing factor in the development of acute corneal hydrops in our case. The presence of cerulean blue dot cataract in the contralateral eye also makes our case distinct. If a patient complains of redness, pain, photophobia, or watering in the eye at all, an ophthalmological evaluation is a must. However, it is imperative to highlight that early presentation of these cases, an accurate diagnosis, and prompt initiation of treatment could prevent these eyes from becoming blind. This case illustrates that children with Down’s syndrome can have a multitude of problems. Beside systemic evaluation, a thorough ocular examination is a must to prevent ocular morbidity and to give them an improved quality of life.

## Conclusion

This is a rare case of both a genetic syndrome and endocrine disease in a child contributing to the development of acute ocular injury. Managing such cases required a multidisciplinary approach including endocrinologist, ophthalmologist, pathologist and paediatrician. Ophthalmologists and non-ophthalmologists should keep mind the rare association of Down’s with hypothyroidism with keratoconus so as to prevent a progression of the disease. This will not only help in salvaging the other eye but also will prevent irreversible visual decline and life-threatening sequelae.

## Notes

### Patient consent 

The authors certify that they have obtained appropriate consent from the patient. In the consent form, the patient has given his consent for his images and other clinical information to be published in the journal. 

### Acknowledgements 

We would like to acknowledge the support of Aravind Eye Hospital and Post Graduate Institute of Ophthalmology, Pondicherry 605007, India.

### Competing interests

The authors declare that they have no competing interests.
